# Material Removal on Hydrogen-Terminated Diamond Surface via AFM Tip-Based Local Anodic Oxidation

**DOI:** 10.3390/mi16090981

**Published:** 2025-08-26

**Authors:** Jinyan Tang, Zhong-Hao Cao, Zhongwei Li, Yuan-Liu Chen

**Affiliations:** 1 The State Key Lab of Fluid Power and Mechatronic Systems, Zhejiang University, Hangzhou 310058, China; jyttang@zju.edu.cn (J.T.); caozh@zju.edu.cn (Z.-H.C.); 2 ZJU-Hangzhou Global Scientific and Technological Innovation Center, Hangzhou 310027, China

**Keywords:** hydrogen-terminated diamond, local anodic oxidation, semiconductor fabrication

## Abstract

Diamond is a promising next-generation semiconductor material, offering a wider band gap, higher electron mobility, and superior thermal conductivity compared with silicon. However, its exceptional hardness makes it challenging to fabricate. In this study, we demonstrate a novel approach to realize material removal on hydrogen-terminated diamond surfaces by atomic force microscope (AFM) tip-based local anodic oxidation. By adjusting both the applied voltage and hydrogen plasma etching parameters, the material is removed over an area larger than the AFM tip size. Notably, the hardness of the material surrounding the removal zone is significantly reduced, enabling it to be scratched with a silicon tip. These findings open a promising pathway for improving the machinability of diamonds in future device applications.

## 1. Introduction

As the semiconductor industry continues to evolve rapidly, there is an increasing demand for advanced materials and processing techniques that can meet the performance requirements of next-generation devices. Diamond has exceptional properties such as a wide band gap, high thermal conductivity, and outstanding electron mobility [1,2,3]. Its potential in semiconductor applications has been recognized, such as in diamond metal-oxide-semiconductor field-effect transistors (MOFETs) [4], quantum sensors [5], and optoelectronic devices [6]. Single-crystal diamond is intrinsically an insulator; when hydrogen-terminated and exposed to ambient air, the top surface layer, with a thickness of less than 10 nm, can exhibit p-type conductivity without intentional doping [7,8]. This surface conductivity is attributed to the formation of a two-dimensional hole gas (2DHG) on the diamond surface [9,10]. Leveraging this property, hydrogen-terminated diamond has been employed in the fabrication of microdevices [9,11]. Nevertheless, the extreme hardness of diamond poses significant challenges for its machining and device integration.

AFM tip-based local anodic oxidation is an effective method to fabricate nanostructures on hard and brittle materials such as silicon [12] and silicon carbide [13]. By post-etching with several etchants, material removal can be realized. Taking advantage of the conductivity of the hydrogen-terminated diamond, AFM tip-based local anodic oxidation was implemented on a diamond surface by researchers. Tachiki et al. first found that protrusions with a height of 2 nm were formed and local insulation was achieved in the area after anodic oxidation modification. Auger electron spectroscopy (AES) shows evidence of the loss of hydrogen termination and increase in oxygen, which proves the occurrence of an oxidation reaction after modification. However, they later found that the protrusions were not the actual structure, but the artifact of the absorbed water layer [14]. Tao Wang et al. created a sub-nanometer insulating layer with no topographic changes on the surface by using a small bias (below 2 V) [15]. This layer was insulating in the direction perpendicular to the surface, but the subsurface conductivity was still retained. C. Toma et al. found that when the bias was larger than 12 V, depressed structures could be created. They explained that this change was caused by local heating occurring due to severe current crowding [16]. Steve A. Yianni et al. realized material removal by proposing a new method called field-emission scanning probe lithography [17]. They applied a large bias from 50 V to 90 V to generate a high electric field and emit high-energy electronics from the tip, and nano-grooves with depths in the range of 4–8 nm and linewidths of 70–150 nm were fabricated [18].

In this work, a new regime of material removal from hydrogen-terminated diamond via tip-based oxidation is reported. In this regime, a layer of material, far exceeding the radius of the AFM tip, was removed from the hydrogen-terminated surface. The thickness of the removed layer was approximately 0.5 nm. Moreover, the hardness of the material surrounding the removal zone was observed to decrease. Based on this phenomenon, by controlling the oxidation positions, adjacent removal zones were connected to achieve large-area material removal.

## 2. Materials and Methods

**Figure 1:**
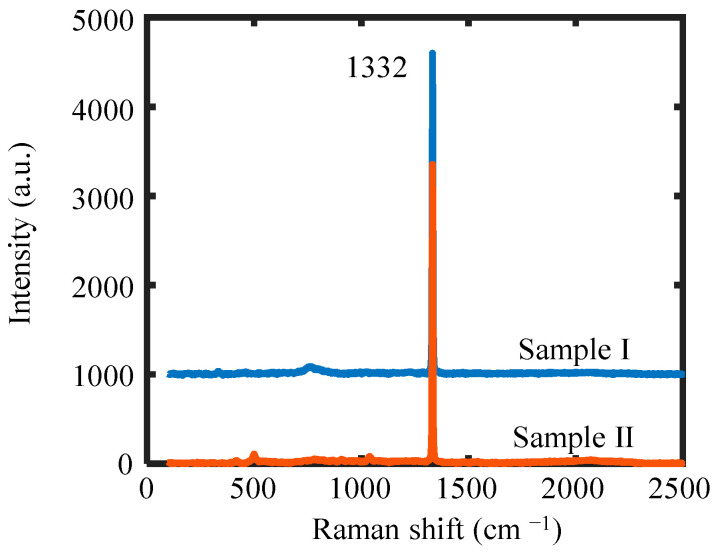
Raman spectra of the two samples after plasma etching.

Two 5 × 5 × 0.5 mm^3^ diamond samples with a (100) crystallographic plane were initially polished to achieve a final roughness of approximately Ra 1 nm. Then, the samples were rinsed in an acidic solution of H_2_SO_4_ and KNO_3_ at 300 °C for 30 min to remove the impurities on the surface. After that, the samples were treated with plasma etching in a microwave-plasma-enhanced chemical vapor deposition (CVD) chamber to obtain hydrogen termination on the surface. The two samples were treated under different conditions, with the detailed parameters provided in Table 1. To prevent the influence of any non-diamond layer formed during the plasma etching process [19], the chamber was pre-cleaned using hydrogen plasma with the parameters shown in Table 1 before the samples were loaded. It should be noted that the C–H bonds on the diamond surface are not very stable in ambient air. The hydrogen termination gradually decreases over several days, accompanied by an increase in surface resistance because of the oxidation by oxygen [20]. In this work, the samples were transported under nitrogen packaging before the experiments to preserve the hydrogen termination. Figure 1 presents the Raman spectra of the two samples after plasma etching. To facilitate the comparison between the two spectra in the same figure, the Raman spectrum of Sample I was shifted vertically by an intensity of 1000. The prominent Raman peak of 1332 cm^−1^ is observed for both samples, which corresponds to the sp^3^ bonding structure of diamond. This indicates that minimal lattice stress remains after plasma etching, as no significant peak shift is observed. The slight difference in intensity between the two spectra may be due to variations in surface roughness after plasma etching. Some minor peaks are observed in the spectrum, which may correspond to some residual defects or surface impurities.

**Figure 2:**
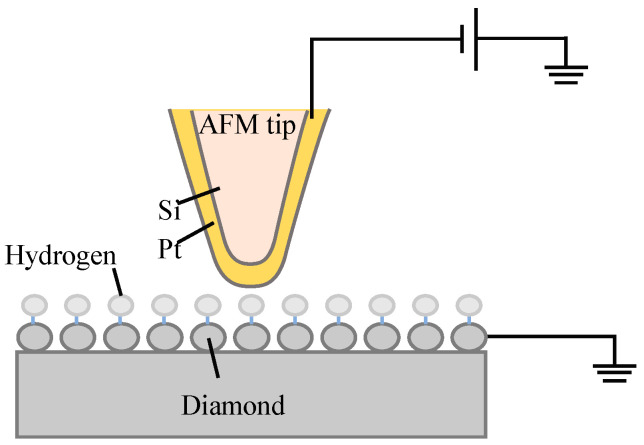
Schematic diagram of the experiment system.

The experiments were performed using a commercial AFM (Dimension Icon, Bruker, Billerica, MA, USA). Conductive silicon tips with platinum coating (HQ:NSC18/Pt, MikroMasch, Sofia, Bulgaria) were employed. The nominal radii of the tips are less than 30 nm. A schematic of the experimental setup is shown in Figure 2. The AFM was operated in contact mode during the experiments. To achieve anodic oxidation on the sample surface, a negative bias was applied to the tip while the sample was grounded, causing the tip to function as the cathode and the sample as the anode. The conductive surface of the sample was connected to a metal wire using conductive silver adhesive, which was then grounded. After the oxidation experiments, the topography of the fabricated structures was imaged with the tapping mode of an AFM, with the same tip used during the oxidation process.

## 3. Results and Discussion

**Figure 3:**
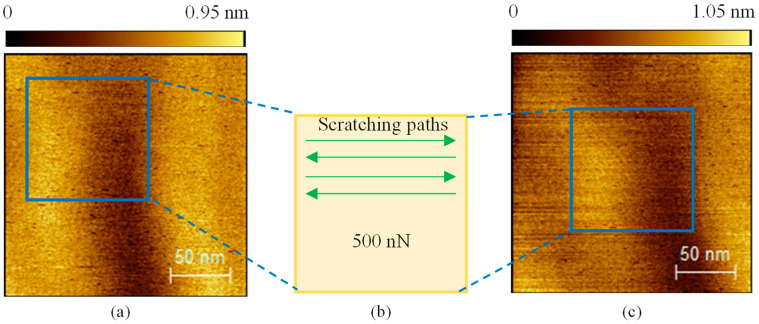
Mechanical scratching testing before local anodic oxidation experiment. (**a**) Original surface before scratching, (**b**) the sketch diagram of the scratching passes, and (**c**) topography of the surface after scratching.

Before the local anodic oxidation experiments, a mechanical scratching test was performed on Sample I as a control experiment to ensure that the mechanical force would not affect the sample surface. As shown in Figure 3, a normal force of approximately 500 nN was applied to the sample surface using the tip, and scratching was conducted within a square area of 100 × 100 nm^2^ at a velocity of 200 nm/s. A total of 256 passes were made. The tip used for scratching was the conductive silicon tip mentioned earlier, the same type used in the subsequent oxidation experiments. After scratching, the sample surface remained nearly unchanged, indicating that mechanical scratching with the silicon tip did not produce any features. To avoid too much tip wear during the oxidation experiments, a reduced normal force of 40 nN was applied.

**Figure 4:**
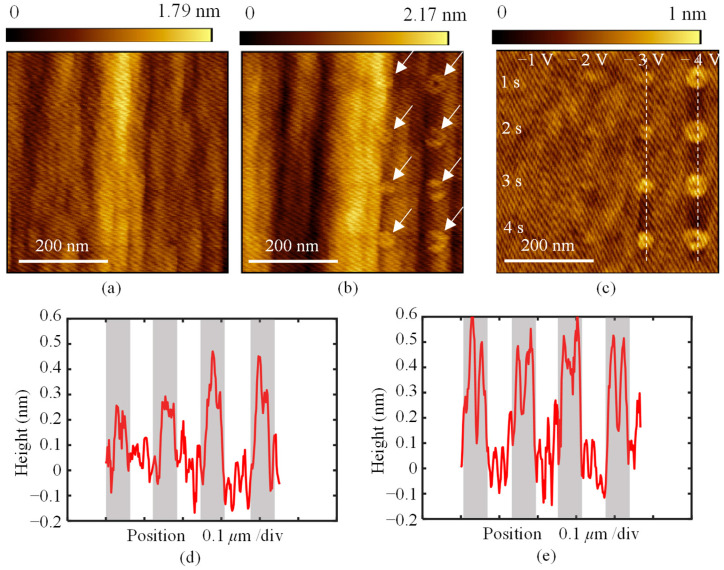
AFM images of the protrusions fabricated on sample Ι. (**a**) The original surface of the diamond. (**b**) Protrusions fabricated on the diamond with various applied bias voltages and durations. (**c**) The topography of the protrusions after the original topography is filtered. (**d**,**e**) are the cross-sectional profiles of the protrusions fabricated with −3 V and −4 V, respectively, marked with the white dashed lines in (**c**).

The local anodic oxidation was first investigated on Sample I, with varying bias voltages applied to the tip and different oxidation durations. The AFM topography images after oxidation are shown in Figure 4. The sample was oxidized with bias voltages ranging from −1 V to −4 V and oxidation durations ranging from 1 s to 4 s. The original surface topography is shown in Figure 4a. In the 500 nm range, the average maximum height of the profile is 1.64 nm. After oxidation, protrusions appeared on the surface, as seen at the position marked by white arrows in Figure 4b. For a clearer view of the distribution and topography of these protrusions, the original topography was filtered, and the results are shown in Figure 4c. When the bias voltage was less than −2 V, no protrusions were observed, even as the duration time increased. However, when the bias voltage exceeded −3 V, protrusions with varying heights were formed. The cross-sectional profiles of the protrusions fabricated at −3 V and −4 V are shown in Figure 4d and 4e, respectively. At a voltage of −3 V, as the oxidation time increased, the height of the protrusions grew from 0.2 nm to 0.4 nm. At −4 V, the protrusions formed had a similar height of approximately 0.5 nm.

Similar results were found by researchers. The modified area was found to have improved hydrophilicity and decreased conductivity [19,21], which is the indication of the reduction in hydrogen termination. An increase in the signature of oxygen was observed at the modified area by scanning auger microprobe analysis, which confirmed the occurrence of oxidation reactions [22]. However, in general, the oxidation of a diamond (100) surface is a process of carbon atoms turning into gas, such as CO and CO_2_, layer by layer. During this process, terminations of C-O and C=O bonds were formed with the surficial carbon atoms [23]. The crystal diamond C (1 × 1) surface was observed by micro reflection high-energy electron diffraction (RHEED) in the oxidized area [24]. Thus, the surface volume does not increase during the oxidation, and the protrusions observed in AFM tapping mode should not be interpreted as real bump structures, unlike those formed in the oxidation of silicon. Given that the hydrophily and resistance of the oxidized area increased greatly, the protrusions have been widely considered as an artifact caused by the absorbed water layer or the increased electrostatic force [25,26,27].

**Figure 5:**
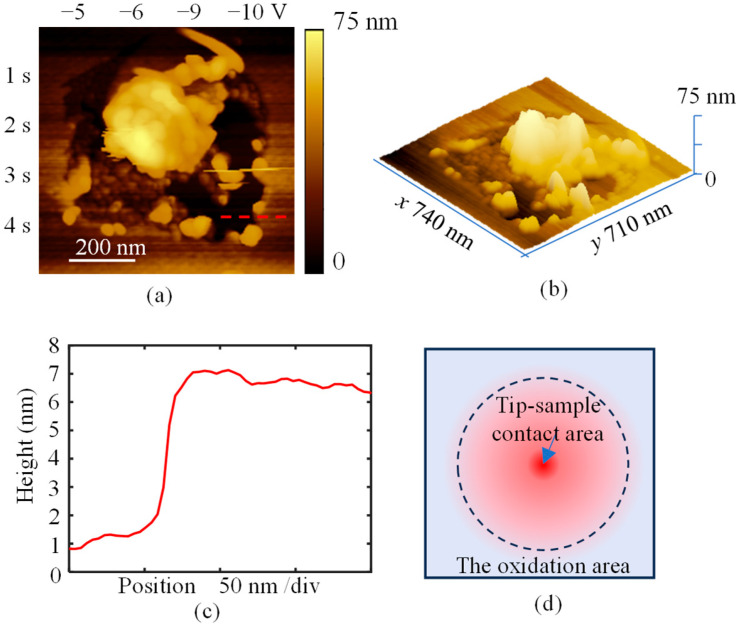
AFM images of the structure on sample Ι after anodic oxidation with the bias of −5 to −10 V. (**a**) The 2D image of the topography after applying different bias voltages and different durations. (**b**) The 3D image of the topography shown in (**a**). (**c**) The cross-sectional profile of the red dashed line shown in (**a**). (**d**) The schematic diagram of the current density distribution around the tip–sample contact area.

When larger bias voltages ranging from −5 V to −10 V were applied to the surface, a large-area depressed structure was formed, as shown in Figure 5a,b. At the edges of the depressed structure, several irregular bumps were observed, while a prominent bump with a height of approximately 40 nm appeared in the center. The depth of the depressed structure was measured at the position marked by the red dashed line in Figure 5a and is shown in Figure 5c to be about 6 nm. It should be noted that this measured depth may overestimate the actual value due to the uneven edges of the structure. The observed large-area material removal may be attributed to the thin conductive layer on the surface. During hydrogen plasma etching, hydrogen atoms can diffuse into the interstitial sites of diamond to a sub-nanometer depth [28], slightly weakening the bond between this layer and the substrate. Under a large bias voltage, the current density in the oxidation area increases, expanding the affected region, and the conductivity in this region decreases after oxidation because the hydrogen termination is replaced by oxygen termination [19,21], as illustrated in Figure 5d. Consequently, heat accumulation in the oxidized region is enhanced due to the high local resistance, which further softens the material [16]. Under the combined effects of heat and oxidation, graphitization or amorphization may occur. This process further weakens the interaction between the conductive layer and the substrate, allowing the modified layer to be detached by the tip.

**Figure 6:**
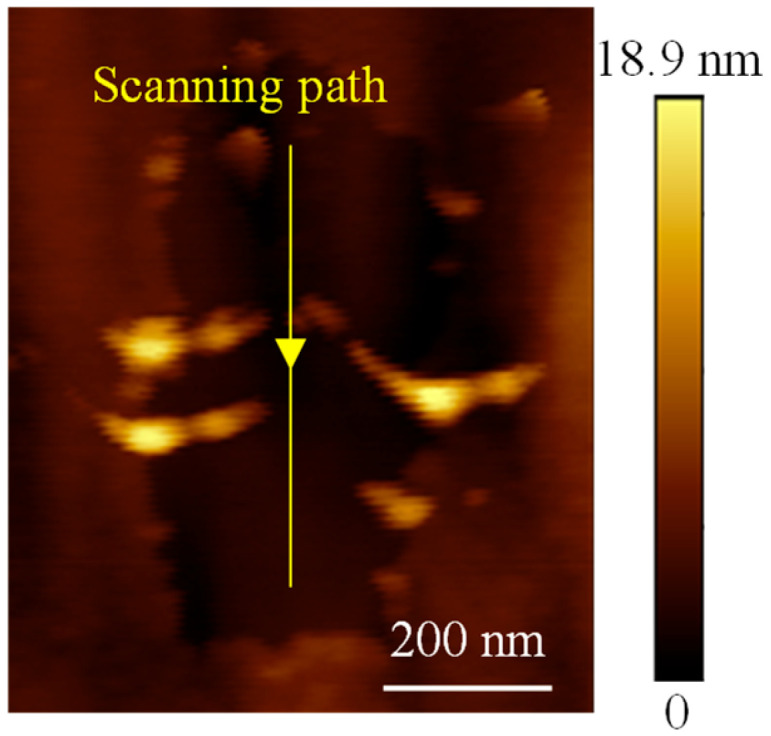
Line oxidation with a bias voltage of −10 V.

This phenomenon is not accidental. To verify its reproducibility, a line oxidation experiment was performed on another sample processed together with Sample I, as shown in Figure 6. During the experiment, a bias voltage of −10 V was applied while the AFM tip scanned at a speed of 100 nm/s. A large area of material removal was observed surrounding the oxidized line, confirming the repeatability of this material removal behavior.

**Figure 7:**
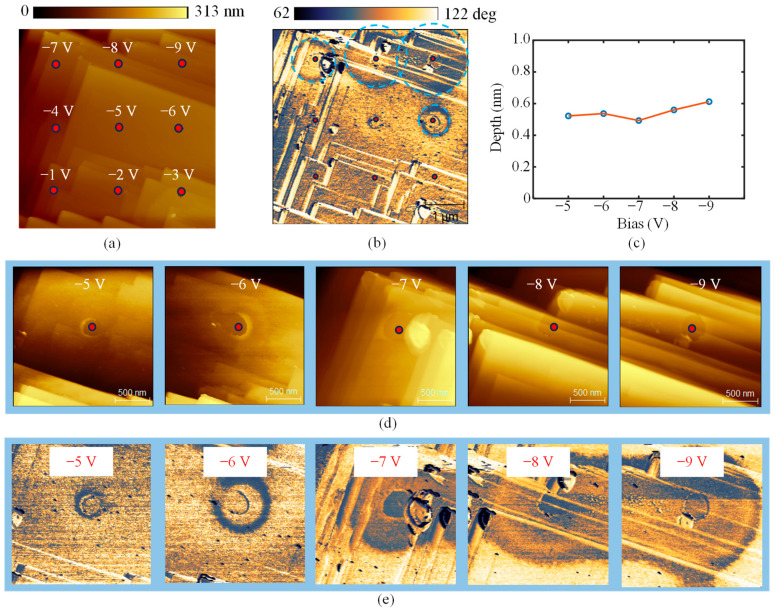
AFM images of the 3 × 3 array of the structures on sample II oxidized with various biases from −1 V to −9 V and a duration of 1 s. (**a**) The topography. (**b**) The phase shift. (**c**) The relationship between the bias and the depressed structures. (**d**,**e**) are the enlarged images of the topography (**d**) and phase shift (**e**) of the depressed structures fabricated with bias from −5 V to −9 V.

Figure 7 illustrates the features fabricated on Sample II. Oxidation was performed on a 3 × 3 array with bias voltages ranging from −1 V to −9 V and a duration of 1 s. Figure 7a,b presents the topography and phase images obtained in the tapping mode of the AFM. No discernible features were observed when the bias voltage was below −5 V. As the bias voltage increased, areas of material removal appeared, accompanied by circular phase shifts. The phase contrast in tapping mode reflects changes in surface properties such as hardness and hydrophobicity [29,30]. Detailed topography and phase images of the oxidation regions under bias voltages from −5 V to −9 V are shown in Figure 7d,e. Both the area of the depressed structures and the corresponding phase-shifted regions increased with applied voltage. At a bias of −9 V, the diameter of the depressed structure reached 340 nm, while the diameter of the phase-shifted area extended to 1.9 μm, far exceeding the tip radius (~30 nm). The substantially larger phase-shifted area indicates that the properties of the material surrounding the contact zone were also altered. The relationship between the depth of the depressed structures and the applied bias voltage, extracted from Figure 7d, is presented in Figure 7c. The depth of the depressed layer remained nearly constant at approximately 0.5 nm with increasing bias voltage, consistent with the conclusion that material removal was confined to the thin etched layer.

**Figure 8:**
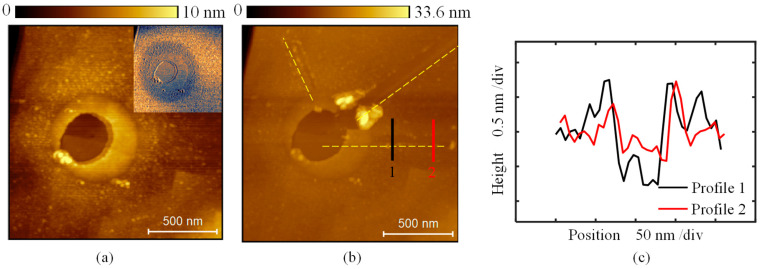
The depressed structure obtained by a bias of −6 V and duration of 3 s. (**a**) The topography. (**b**) Three nano-grooves were fabricated by mechanical scratching using a silicon tip. (**c**) The cross-sectional profile of the nano-groove marked in (**b**).

To investigate the changes in surface properties, three mechanical scratches were applied on the depressed structure fabricated with a bias of −6 V and a duration of 3 s, as shown in Figure 8. Figure 8a presents the topography after oxidation, where a depressed structure with a diameter of 390 nm was created at the center. The raised edge surrounding the depressed structure reaches approximately 3 nm in height. The inset shows the phase shift image, indicating that the surface properties of a large area surrounding the depressed structure have changed. Figure 8b shows three nano-grooves fabricated on the diamond surface with a normal force of 200 nN using the same silicon tip employed in the oxidation experiment. The cross-sectional profiles along the two positions marked in Figure 8b are presented in Figure 8c. The depth of the nano-grooves decreases from 1 nm at the center to 0.5 nm toward the outer region of the oxidation area. This mechanical scratching results indicate that after a large area of material was removed by oxidation, the hardness around the oxidation site decreased compared with the original surface. By comparing the depths of the two cross-sectional profiles in Figure 8c, it is evident that the hardness decreases gradually from the outer edge toward the center of the oxidation area.

**Figure 9:**
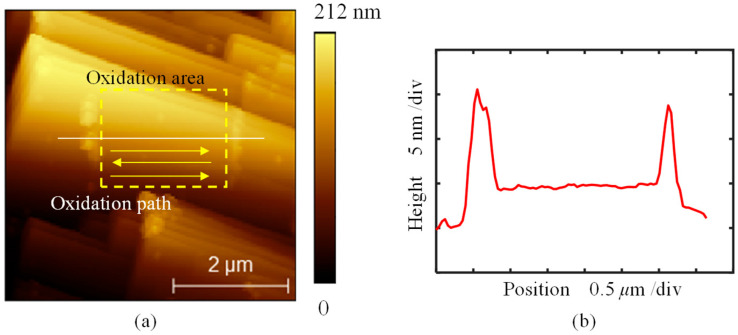
Topography obtained by the AFM tapping mode after continuous oxidation. (**a**) The topography of the oxidation area. (**b**) The cross-sectional profile marked with a white solid line in (**a**).

Then, oxidation was performed to form a continuous surface with a bias of −6 V, as shown in Figure 9. The oxidized area measured 2 μm × 2 μm, and the tip scanned along the path illustrated in Figure 9a. The tip moved at 400 nm/s along the fast axis, completing 512 passes along the slow axis. The cross-sectional profile of the oxidized surface is shown in Figure 9b, where the height of the surface increased by 4.1 nm relative to the surrounding area. Compared with the spot oxidation shown in Figure 7 and Figure 8, no large-area material removal was observed, even under the same bias voltage. This difference arises from the more distributed heat and oxidation current during continuous scanning. The formation of protrusions is attributed to artifacts caused by the adsorbed water layer or increased electrostatic force [26,27], which is consistent with the origin of the dot-like protrusions observed in Figure 4. Additionally, some material accumulation was observed at the edges of the oxidized area. Since this continuous oxidation was conducted after the spot oxidation experiments in Figure 7 and Figure 8, some previously removed material adhered to the tip and was transferred along at the end of the tip path during this continuous oxidation.

**Figure 10:**
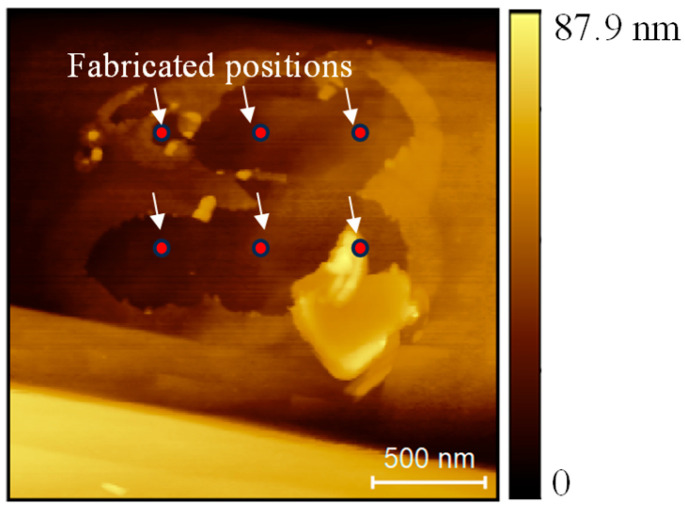
A 2 × 3 array oxidized with a voltage of −6 V and duration of 3 s.

By adjusting the oxidation positions, the depressed structures were connected, as shown in Figure 10. Oxidation was performed on a 2 × 3 array with a bias voltage of −6 V and a duration of 3 s. The spacing between the adjacent positions was 500 nm. After oxidation, a material layer with an area of approximately 1.5 μm × 1 μm was removed. Moreover, a large fragment of the removed material can be observed adjacent to the fabricated regions, providing additional evidence that the material was softened and detached by the tip. This approach could be extended to larger areas by applying a tool with larger size, enabling efficient removal of the surface layer of hydrogen-terminated diamond and showing potential for application in diamond surface polishing. In addition, this method allows the facile formation of selective high-resistance regions at the micrometer scale, which may be exploited in electronic device applications [31] because the method can realize material removal over an area larger than the AFM tip size, resulting in higher efficiency compared with conventional local anodic oxidation on semiconductors such as silicon [12], where the oxidation is typically confined to a region comparable to the tip size.

In our future work, we will establish an electrochemical detection system for AFM tip-based local anodic oxidation, enabling real-time monitoring of the oxidation process. This system will allow us to systematically investigate the underlying electrochemical mechanisms, including cyclic voltammetry characteristics, current response dynamics, and peak potential variations. The insights gained from these studies are expected to provide a deeper understanding of the tip-induced electrochemical processes on the hydrogen-terminated diamond and contribute to the development of more precise and controllable nanoscale fabrication techniques.

## 4. Conclusions

In this study, a new regime of material removal on hydrogen-terminated diamond, based on local anodic oxidation and localized heating, was investigated. A material layer with an area far exceeding the size of the AFM tip can be removed by the oxidation process when the applied voltage exceeds −5 V. For instance, at a bias voltage of −9 V and a duration of 1 s, a depressed structure with a diameter of 340 nm was created using a tip with a diameter of only 30 nm. Meanwhile, the material surrounding the oxidation area became softer, with hardness gradually increasing along the radial direction. Highly localized heating plays a critical role in forming these large-area depressed structures. By connecting the removed areas, the surface material of hydrogen-terminated diamond can be efficiently removed, providing a potential approach to enhance the machinability of diamond.

## Figures and Tables

**Table 1:** The conditions of plasma etching for the two samples.

Conditions	Chamber Cleaning	Sample I	Sample II
Temperature (°C)	850	800	800
Gas pressure (Torr)	80	80	80
Microwave power (W)	2500	3000	2500
Etching time (min)	90	60	180
Gas flow (sccm)	1000	1000	100

## Data Availability

Data underlying the results presented in this paper are available from the authors upon request.

## Abbreviations

The following abbreviations are used in this manuscript:

AFM	atomic force microscope
MOFET	metal-oxide-semiconductor field-effect transistor (MOFET)
2DHG	2D hole gas
CVD	chemical vapor deposition (CVD)
RHEED	reflection high-energy electron diffraction (RHEED)
